# Marked Cortisol Production by Intracrine ACTH in GIP-Treated Cultured Adrenal Cells in Which the GIP Receptor Was Exogenously Introduced

**DOI:** 10.1371/journal.pone.0110543

**Published:** 2014-10-21

**Authors:** Hiroko Fujii, Mimi Tamamori-Adachi, Kousuke Uchida, Takao Susa, Takashi Nakakura, Haruo Hagiwara, Masayoshi Iizuka, Hiroko Okinaga, Yuji Tanaka, Tomoki Okazaki

**Affiliations:** 1 Department of General Medicine, National Defense Medical College, Tokorozawa City, Saitama, Japan; 2 Department of Biochemistry, Teikyo University School of Medicine, Tokyo, Japan; 3 Department of Anatomy, Teikyo University School of Medicine, Tokyo, Japan; 4 Department of Internal Medicine, Teikyo University School of Medicine, Tokyo, Japan; Georgia Regents University, United States of America

## Abstract

The ectopic expression of the glucose-dependent insulinotropic polypeptide receptor (GIPR) in the human adrenal gland causes significant hypercortisolemia after ingestion of each meal and leads to Cushing’s syndrome, implying that human GIPR activation is capable of robustly activating adrenal glucocorticoid secretion. In this study, we transiently transfected the human GIPR expression vector into cultured human adrenocortical carcinoma cells (H295R) and treated them with GIP to examine the direct link between GIPR activation and steroidogenesis. Using quantitative RT-PCR assay, we examined gene expression of steroidogenic related proteins, and carried out immunofluorescence analysis to prove that forced GIPR overexpression directly promotes production of steroidogenic enzymes CYP17A1 and CYP21A2 at the single cell level. Immunofluorescence showed that the transfection efficiency of the GIPR gene in H295R cells was approximately 5%, and GIP stimulation enhanced CYP21A2 and CYP17A1 expression in GIPR-introduced H295R cells (H295R-GIPR). Interestingly, these steroidogenic enzymes were also expressed in the GIPR (–) cells adjacent to the GIPR (+) cells. The mRNA levels of a cholesterol transport protein required for all steroidogenesis, StAR, and steroidogenic enzymes, HSD3β2, CYP11A1, CYP21A2, and CYP17A1 increased 1.2-2.1-fold in GIP-stimulated H295R-GIPR cells. These changes were reflected in the culture medium in which 1.5-fold increase in the cortisol concentration was confirmed. Furthermore, the levels of adenocorticotropic hormone (ACTH) receptor and ACTH precursor proopiomelanocortin (POMC) mRNA were upregulated 2- and 1.5-fold, respectively. Immunofluorescence showed that ACTH expression was detected in GIP-stimulated H295R-GIPR cells. An ACTH-receptor antagonist significantly inhibited steroidogenic gene expression and cortisol production. Immunostaining for both CYP17A1 and CYP21A2 was attenuated in cells treated with ACTH receptor antagonists as well as with POMC siRNA. These results demonstrated that GIPR activation promoted production and release of ACTH, and that steroidogenesis is activated by endogenously secreted ACTH following GIP administration, at least in part, in H295R cells.

## Introduction

Glucose-dependent insulinotropic polypeptide (GIP) is a 42 amino acid peptide hormone released from intestinal K cells upon nutrient ingestion. GIP exerts multiple biological effects via GIP receptor (GIPR), which is a G-protein-coupled receptor (GPCR), through cAMP production, resulting in glucose-stimulated insulin production and secretion, cell proliferation, and anti-apoptosis in pancreatic beta-cells [1], [2].

Adenocorticotropic hormone (ACTH) is a physiological modulator of steroidogenesis in the adrenal cortex. Binding to its receptor, melanocortin 2 receptor (MC2R), activates adenylyl cyclase and leads to cAMP production with cAMP-dependent protein kinase A (PKA) activation and phosphorylation of specific transcriptional factors, which regulate free cholesterol availability and activate steroidogenic enzyme expression [3]–[11]. Several studies have shown that hyperplastic adrenal glands display abnormal expression of aberrant receptors including GPCRs involved in the control of cortisol secretion. The ectopic expression of these receptors functionally coupled to steroidogenesis confers inappropriate sensitivity on adrenocortical cells to either GIP, catecholamines or other hormones (angiotensin II, glucagon, serotonin 5HT_7_, thyrotropin, luteinizing hormone, V_2_-vasopressin etc). The underlying pathophysiology has been thought to be independent of ACTH [12]–[19]. Surprisingly, Louiset *et al.* recently reported that cortisol secretion from the adrenal glands of patients with macronodular hyperplasia of Cushing’s syndrome appears to be regulated by ACTH, which is produced by a subpopulation of steroidogenic cells in the hyperplastic adrenal glands, but not by pituitary adrenocorticotroph cells. Tissues containing aberrant GPCRs release ACTH and cortisol during perifusion with GIP, serotonin, or human chorionic gonadotropin. The ACTH-receptor antagonist ACTH (7–38) inhibits cortisol secretion by 40% in these tissues. Thus, they showed that cortisol production is apparently controlled dually by aberrant GPCRs and by ACTH produced within the adrenocortical tissue, amplifying the effect of the aberrant receptors [20].

The ectopic expression of GIPR in the human adrenal gland causes significant hypercortisolemia after ingestion of a meal and leads to food-dependent Cushing’s syndrome (FD-CS), demonstrating that activation of human GIPR is capable of robustly activating adrenal glucocorticoid secretion [21]–[25]. Indeed, GIP administration increases corticosterone levels in rats, and isolated rat adrenocortical zona fasciculate/reticularis cells respond to GIP in a cAMP-dependent manner [15]. Mazzuco *et al.* reported that bovine adrenal cells transfected with the GIPR and injected under the renal capsule of mice lead to the development of hyperplastic adrenal glands and hypercortisolism [26]. Drucker’s group reported that GIP stimulates cAMP production and steroidogenic gene expression using mouse Y1 cells stably expressing GIPR [27]. Thus, several indirect sources of evidence demonstrate that GIP promotes cAMP activation via GIPR, followed by steroidogenesis in adrenocortical cells. However, the detailed nexus between activated GIPR and steroidogenesis, especially in humans, is largely unknown.

The aim of our study was to investigate whether activated GIPR mediates ACTH secretion and steroidogenesis, and whether GIPR-induced steroidogenesis is operated through secreted ACTH in adrenocortical cells. H295R cells are transformed human adrenal cells that secrete all of the steroid intermediates of the steroidogenesis pathway, and have been found useful for studying cAMP/PKA signaling and steroidogenesis in adrenocortical cells [28]–[31]. In this study, we transiently transfected human GIPR gene to H295R cells, treated them with GIP, and investigated the expression of ACTH and its involvement in steroidogenesis promoted by activated GIPR in these cells.

## Materials and Methods

### Ethics Statement

The protocol for the collection of adrenal samples and the experimental procedures were approved by Ethical Review Committee of National Defense Medical College (No. 773). Written informed consent was obtained from all patients.

### Materials

C-terminally FLAG-tagged GIPR was PCR-amplified using human GIPR cDNA (Clone ID: #7939568; Open Biosystems). The primer set was as follows, 5′-ATACTCGAGGCCACCATGACTACCTCTCCGATCCTGCAGCTGC-3′ (forward) and 5′-ATACTCGAGCTACTTGTCATCGTCGTCCTTGTAGTCGCAGTAACTTTCCAACTCCCGGCT-3′ (reverse) that encoded FLAG-tag sequence. The PCR product was subcloned into pcDNA3.1Zeo (+) at EcoRV site. Eight-bromoadenosine 3′,5′-cyclic monophosphate (8-Br-cAMP) and forskolin were purchased from Sigma-Aldrich (St Louis, MO, USA). GIP was purchased from Peptide Institute Inc (Osaka, Japan). ACTH (7–38) was purchased from Wako Pure Chemical Industries, Ltd (Osaka Japan). H-89 dihydrochloride was purchased from Calbiochem (San Diego, CA, USA). The following antibodies were used in this study: primary antibodies: anti-mouse FLAG M2 monoclonal (F1804; Sigma-Aldrich), anti-rabbit GIPR monoclonal (ab124939; Abcam plc, Cambridge, UK), anti-goat CYP21A2 polyclonal (C-17) (sc-48466, Santa Cruz, Santa Cruz, CA), anti-rabbit CYP17A1 polyclonal (ab80206; Abcam) antibodies, and secondary antibodies: Alexa Fluor 647 goat a anti-mouse/rabbit IgG (H+L) (A-21236/A-21245; Molecular Probes, Eugene, OR, USA), Alexa Fluor 647 donkey anti-goat IgG (H+L) (A-21447, Molecular Probes), Alexa Fluor 546 goat anti-mouse IgG (H+L) (A-11030, Molecular Probes), Alexa Fluor 546 donkey anti-mouse/rabbit IgG (A-10036/A-10040; Molecular Probes). Anti-rabbit ACTH (1–39) polyclonal antibody was given by S. Tanaka [32].

### Cell culture

Human adrenal cortical cells (NCI-H295R pluripotent adrenocortical carcinoma cell line) were purchased from American Type Culture Collection (ATCC, Manassas, VA, USA). The cells were cultured in DMEM/F-12K (1∶1) (ATCC) supplemented with 1% ITS+Premix (final concentrations of 6. 25 µg/ml insulin/transferrin, 6.25 ng/ml selenium, 1.25 mg/ml bovine serum albumin, and 5.35 µg/ml linoleic acid) (BD Biosciences, Bedford, MA, USA), 2.5% NuSerum (NuSerum containing with 25% New born Calf Serum) (BD Biosciences), and Penicillin/Streptomycin (100 U/ml penicillin and 0.1 mg/ml streptomycin) (GIBCO BRL, Palo Alto, CA), at 37°C in a humidified atmosphere containing 5% CO_2_. The serum-free starvation medium consisted of DMEM/Ham’s F-12 medium, and penicillin/streptomycin. The cells for experiments were plated in 6-well plates at a density of 6.0×10^5^ cells per well and cultured in the growth medium for 24 h.

### 8-Br-cAMP and forskolin treatment

For the RT-PCR experiments, the cells were cultured in the starvation medium for 24 h and then treated with 8-Br-cAMP (500 µM) and forskolin (10 µM) for 6 h. For the immunofluorescent experiments and cortisol measurements, the cells were cultured in the growth medium for 24 h, and then treated with 8-Br-cAMP and forskolin for 48 h.

### Transient transfection and GIP treatment

One day after plating, pcDNA3.1Zeo (+) (empty vector) or pcDNA3.1Zeo (+)-FLAG-tagged human GIPR vector (the hGIPR-c’FLAG) was introduced into H295R cells using Lipofectamine 2000 Reagent (Invitrogen, Carlsbad, CA, USA). For the RT-PCR experiments, at 24 h after transfection, the medium was changed to the starvation medium. The cells were then incubated for another 24 h, and followed by the treatment with 10^−7^ M human GIP for 6 or 24 h. For the immunofluorescent experiments and cortisol assays, at 24 h after transfection, the medium was changed to the growth medium, and on the next day, the cells were treated with 10^−7^ M human GIP for 48 h.

### siRNA transfection

Control siRNA and mixtures of siRNAs for the ACTH precursor proopiomelanocortin (POMC) were obtained from Dharmacon/ThermoFisher Scientific, Inc. (Waltham, MA, USA). siRNAs were co-transfected with the hGIPR-c’FLAG vector using Lipofectamine 2000 according to the manufacturer’s instructions.

### Cortisol assays

Cortisol production from H295R cells and adrenal cells from patients was analyzed by measuring its concentration in culture medium using a Chemiluminescent Enzyme Immunoassay (LSI Medience corporation). Three independent experiments were performed in triplicate, and followed by statistical analyses.

### Reverse-transcription and real-time quantitative PCR

Total RNA was extracted with RNA iso-Plus (TaKaRa Bio Inc. Shiga, Japan) according to the manufacturer’s instructions. Total RNA (1 µg) was treated with 122.5 U DNase I (Invitrogen) in a 100 µl reaction for 1 hour at 37°C. The enzyme was denatured at 90°C for 10 minutes, and then 2.5 µl of the solution was added to each reaction tube. Quantitative RT-PCR (qRT-PCR) was performed using a One Step SYBR PrimeScript RT-PCR kit Ver. 1 (TaKaRa, RR066A) and a Thermal Cycler Dice Real –Time System (TaKaRa, TP800) according to the manufacturer’s instructions. The thermal cycling conditions consisted of an initial denaturation step at 95°C for 30 seconds followed by 40 cycles of PCR under the following conditions: 95°C for 5 s and 60°C for 60 s. *GAPDH* was used as an internal control because this gene, along with the cyclophilin gene, was widely employed as an internal control for the changes of mRNA levels of the steroidogenic enzyme genes [27], [33], [34]. Our choice of the appropriately distant primer sets and the experiments using with or without reverse transcriptase excluded the possibility that our real-time RNA quantification counted genomic DNA. The relative amount of each transcript was calculated with the 2^−ΔΔCt^ method [35] using the cycle threshold value, which was automatically determined by the real-time PCR system by means of the second derivative maximum method [36]. Primer pairs were subsequently tested for performance: absence of primer dimers, and efficiency of amplification >95%, <105%. The primer sets are described in Table S1. Three independent experiments were performed in triplicate, and followed by statistical analyses.

### Immunofluorescence analysis

For immunofluorescence analysis, the cells were cultured on coverslips in 6-well plates. The cells were fixed with 4% paraformaldehyde and 4% sucrose in phosphate-buffered saline (PBS) for 20 min at room temperature. Permeabilization was carried out with 0.25% Triton X-100 in PBS for 5 min at room temperature. Nonspecific binding was blocked by incubation in 10% bovine serum albumin and 0.1% Triton X-100 in PBS for 30 min at 37°C. The antibodies were diluted in the above blocking solution at the indicated concentrations and incubated for 2 h at 37°C. Secondary antibodies were also diluted in the blocking solution and incubated for 30 min at 37°C. Nuclei were stained with 2 µg/ml 4′6-diamidine-2′-phenylindole dihydrochloride (DAPI) (Roche, Mannheim, Germany) in PBS for 15 min at room temperature. Images were acquired using a laser-scanning confocal image system (A1R-A1 Confocal Microscope System; Nikon, Japan). The primary antibodies, anti-flag, anti-GIPR, anti-CYP17A1, anti-CYP21A2, and anti-ACTH were used at concentrations of 1∶1000, 1∶50, 1∶250, 1∶50, 1∶100 respectively, and 2^nd^ antibodies were used at a concentration of 1∶500.

### Statistical analysis

Quantitative data are expressed as the mean ± standard error (SE). Statistical analysis was performed by Student’s *t* test, or one way ANOVA followed by post-hoc Tukey using JMP9 software.

## Results

### Induction of steroidogenesis by 8-Br-cAMP and forskolin in H295R cells

**Figure 1:**
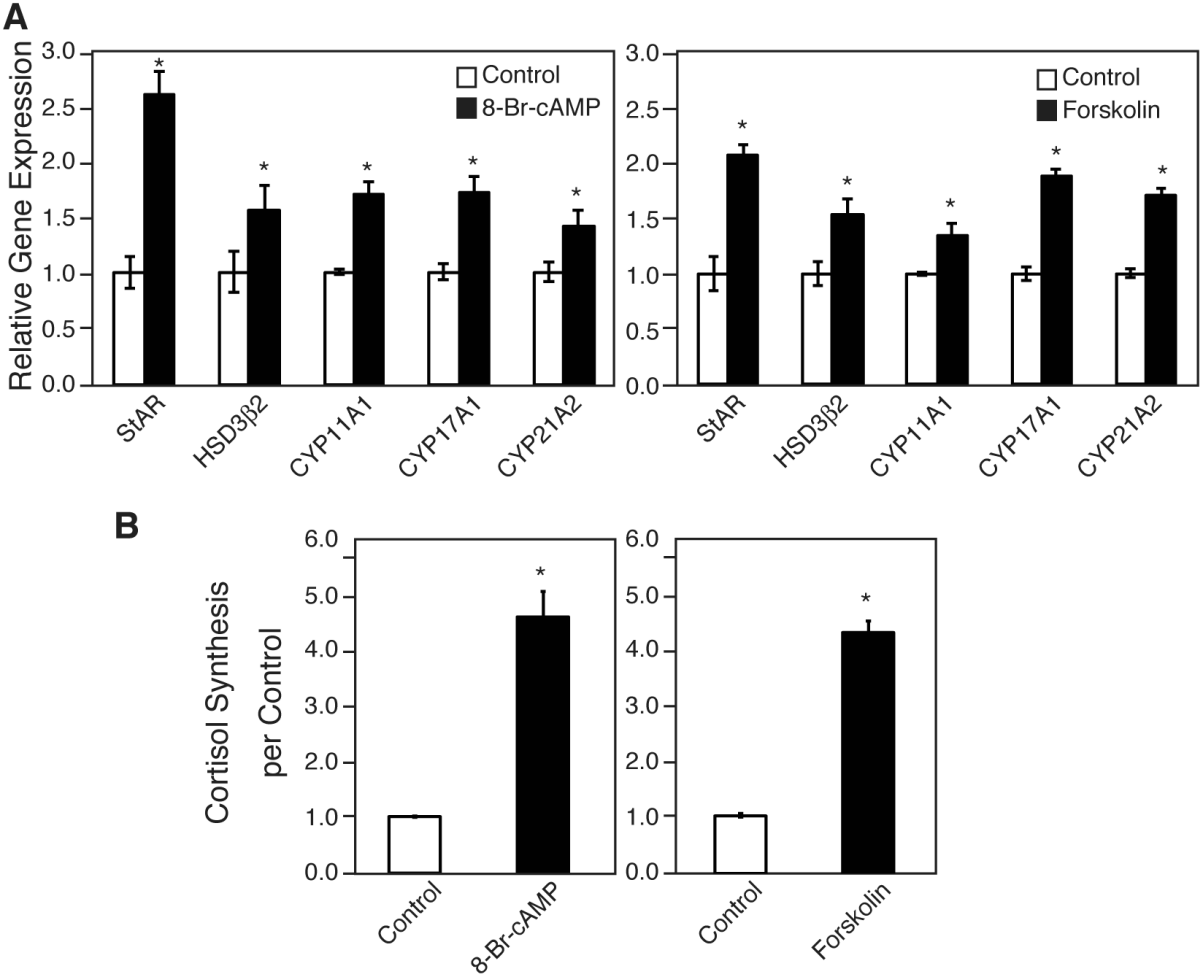
Effects of 8-Br-cAMP and forskolin on the expression of steroidogenesis-related genes and cortisol production. (A) Relative mRNA expression of the indicated genes was analyzed by qRT-PCR. RNA was extracted from H295R cells treated with 8-Br-cAMP (500 µM) or forskolin (10 µM) for 6 h under serum-free conditions (in starvation medium). Data are presented as mean ± SE of three independent experiments. (B) Cortisol concentration in the culture medium of H295R cells. Cortisol synthesis was assessed by the measurement of cortisol concentration in the culture medium of H295R cells treated with 8-Br-cAMP or forskolin for 48 h under growth conditions (in growth medium). Data are presented as mean ± SE of three independent experiments. *P<0.05 vs. control.

**Figure 2:**
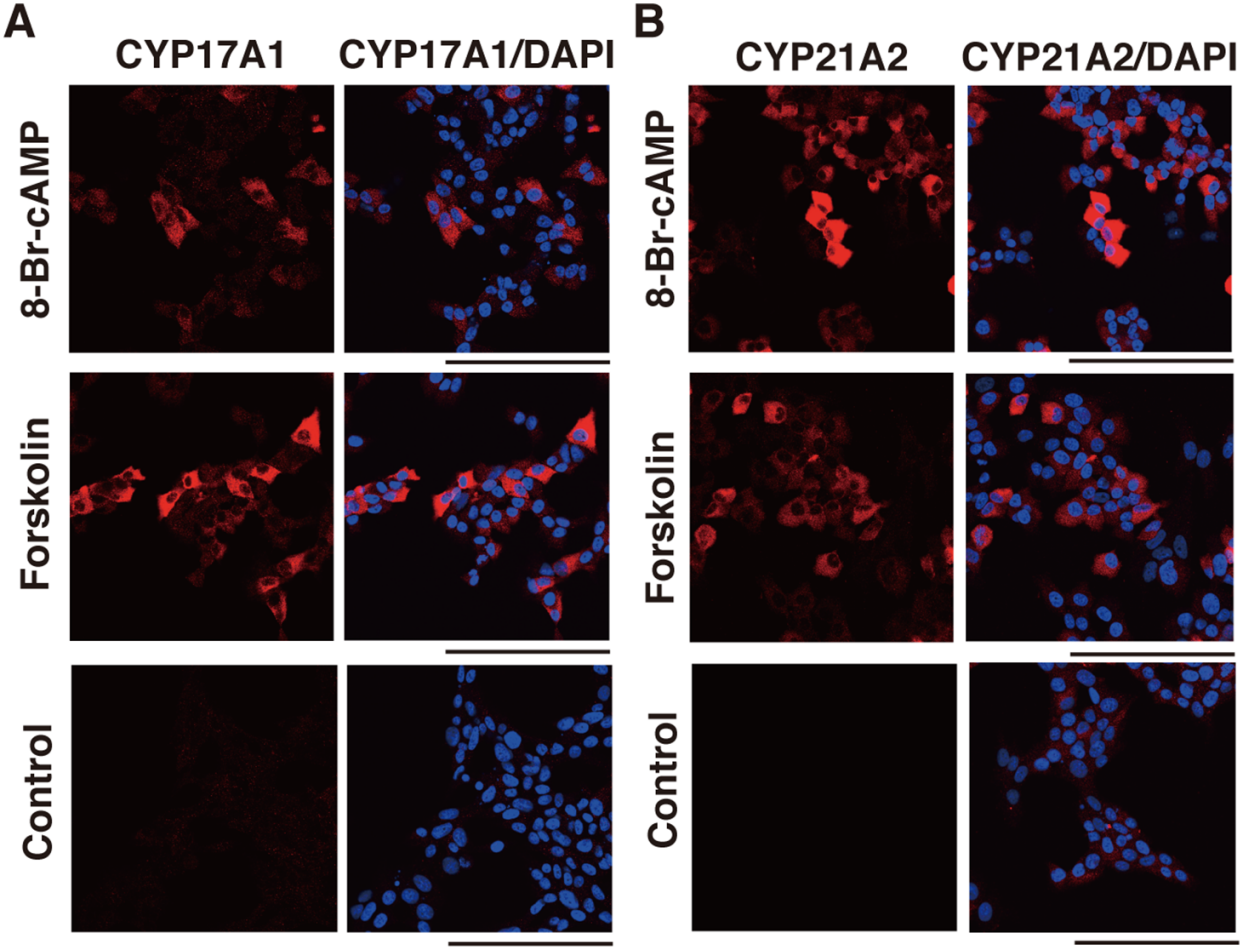
Effects of 8-Br-cAMP and forskolin on the expression of CYP17A1 and CYP21A2. H295R cells were treated with 8-Br-cAMP or forskolin for 48 h under growth conditions, and fixed with 4% paraformaldehyde. (A) Immunostaining for CYP17A1. Red staining shows the anti-CYP17A1 antibody and blue staining shows DAPI (cell nuclei). (B) Immunostaining for CYP21A2. Red staining shows the anti-CYP21A2 antibody and blue staining shows DAPI (cell nuclei). Scale bars represent 100 µm.

To determine whether cAMP activation induces steroidogenic gene expression, we treated H295R cells with 8-Br-cAMP, a cAMP analog and PKA activator, and forskolin, a direct activator of adenylyl cyclase. Quantitative RT-PCR (qRT-PCR) experiments were performed using H295R with or without 8-Br-cAMP or forskolin for 6 h in the starved condition. The levels of StAR, HSD3β2, CYP11A1, CYP17A1, and CYP21A2 mRNA transcripts were significantly increased by 8-Br-cAMP to 2.6-, 1.6-, 1.7-, 1.7-, and 1.4-fold, respectively (Fig. 1A, left panel, p<0.05). Likewise, forskolin increased the levels of StAR, HSD3β2, CYP11A1, CYP17A1, and CYP21A2 mRNA transcripts 2.1-, 1.5-, 1.3-, 1.9-, and 1.7 fold respectively (Fig. 1A, right panel, p<0.05). Further, to examine whether those steroidogenic enzymes are induced at the protein level, we performed immunofluorescence analysis. As shown in Fig. 2, CYP17A1 and CYP21A2 accumulated in the cytoplasm of approximately 30% of cells treated with 8-Br-cAMP or forskolin, whereas control cells expressed neither CYP17A1 nor CYP21A2. We then measured cortisol concentration in the medium of the cells to ensure cortisol production in response to treatment with these reagents. Both 8-Br-cAMP- and forskolin increased cortisol synthesis approximately 4.5-fold (Fig. 1B).

### Induction of steroidogenesis by GIPR activation in H295R cells

**Figure 3:**
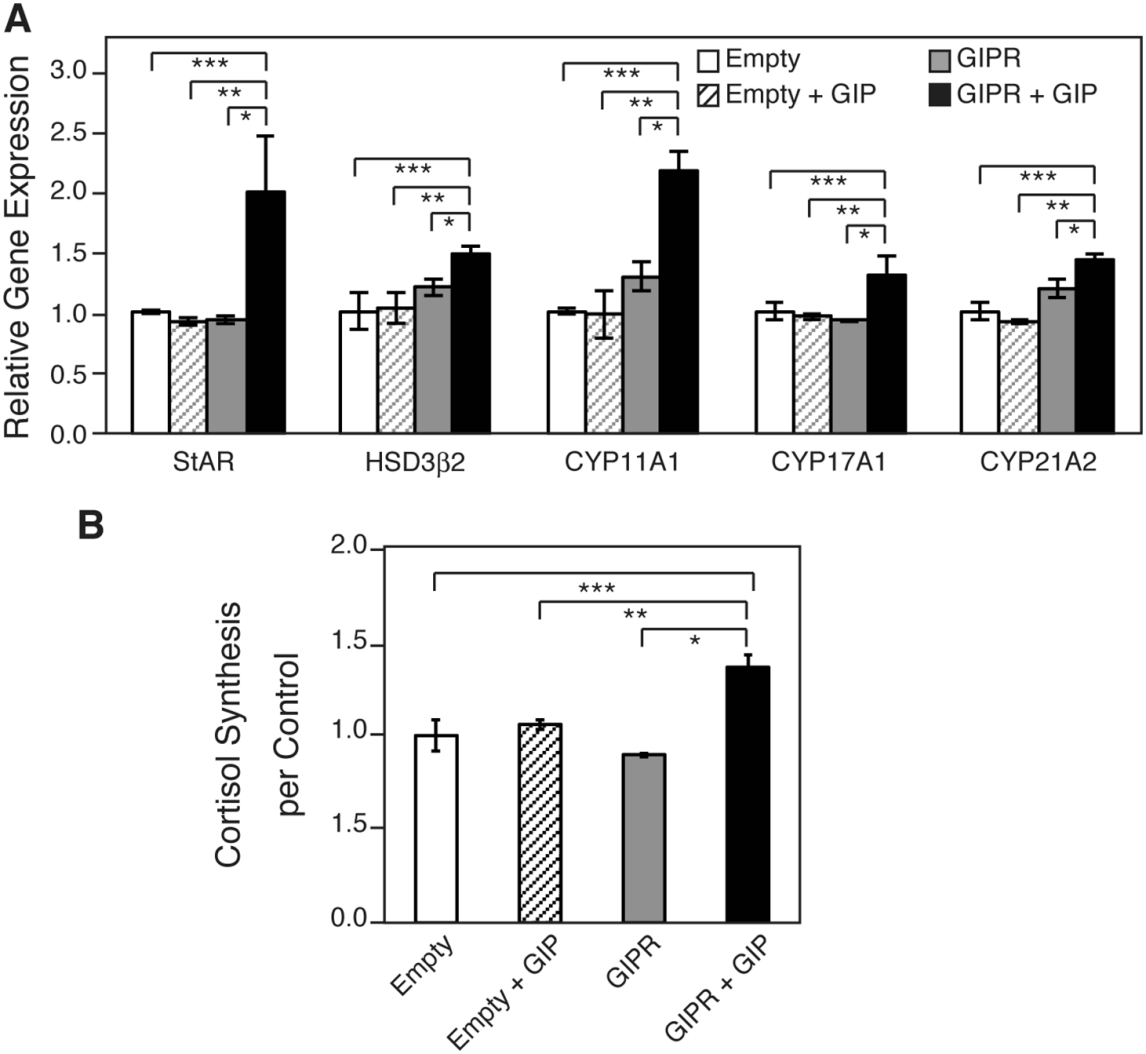
Effects of GIPR activation on the expression of steroidogenesis-related genes and cortisol production. H295R cells were transiently transfected with the empty vector or human GIPR expression vector. (A) Relative mRNA expression of the indicated genes was analyzed by qRT-PCR. At 24 h after transfection, the culture medium was changed to the starvation medium. After 24 h, the cells were treated with or without GIP (10^−7^ M) for 6 h, and following this, RNA was extracted. Data are presented as mean ± SE of three independent experiments. (B) Cortisol concentration in the medium. At 48 h after transfection, the cells were stimulated with or without GIP (10^−7^ M) for 48 h in growth medium. Data are presented as mean ± SE of three independent experiments. *P<0.05 vs. GIPR, **P<0.05 vs. empty + GIP, ***P<0.05 vs. empty.

Having obtained evidence that cAMP activation induced steroidogenesis in H295R cells, we asked whether steroidogenesis is induced by GIP in GIPR-expressing H295R. We transiently introduced the human GIPR gene (hGIPR-c’FLAG vector) into H295R cells and treated them with GIP. The existence of both GIPR- and FLAG-positive cells in cells transfected with the hGIPR-c’FLAG vector was confirmed by immunofluorescence analysis with double staining of anti-FLAG and anti-GIPR antibodies (Fig. S1). FLAG-positive cells comprised approximately 5% of the total number of cells. The rate of FLAG-positive cells was not significantly different between the cells treated with and without GIP. After a 6h-incubation with or without GIP, the H295R-GIPR or H295R-empty cells were harvested, extracted to obtain mRNA, and gene expression was analyzed using qRT-PCR. The levels of StAR, HSD3β2, CYP11A1, CYP17A1, and CYP21A2 mRNA transcripts were significantly increased by GIP to 2.1-, 1.2-, 1.7-, 1.4-, and 1.2-fold, respectively, in the GIPR-transfected H295R cells (Fig. 3A).

**Figure 4:**
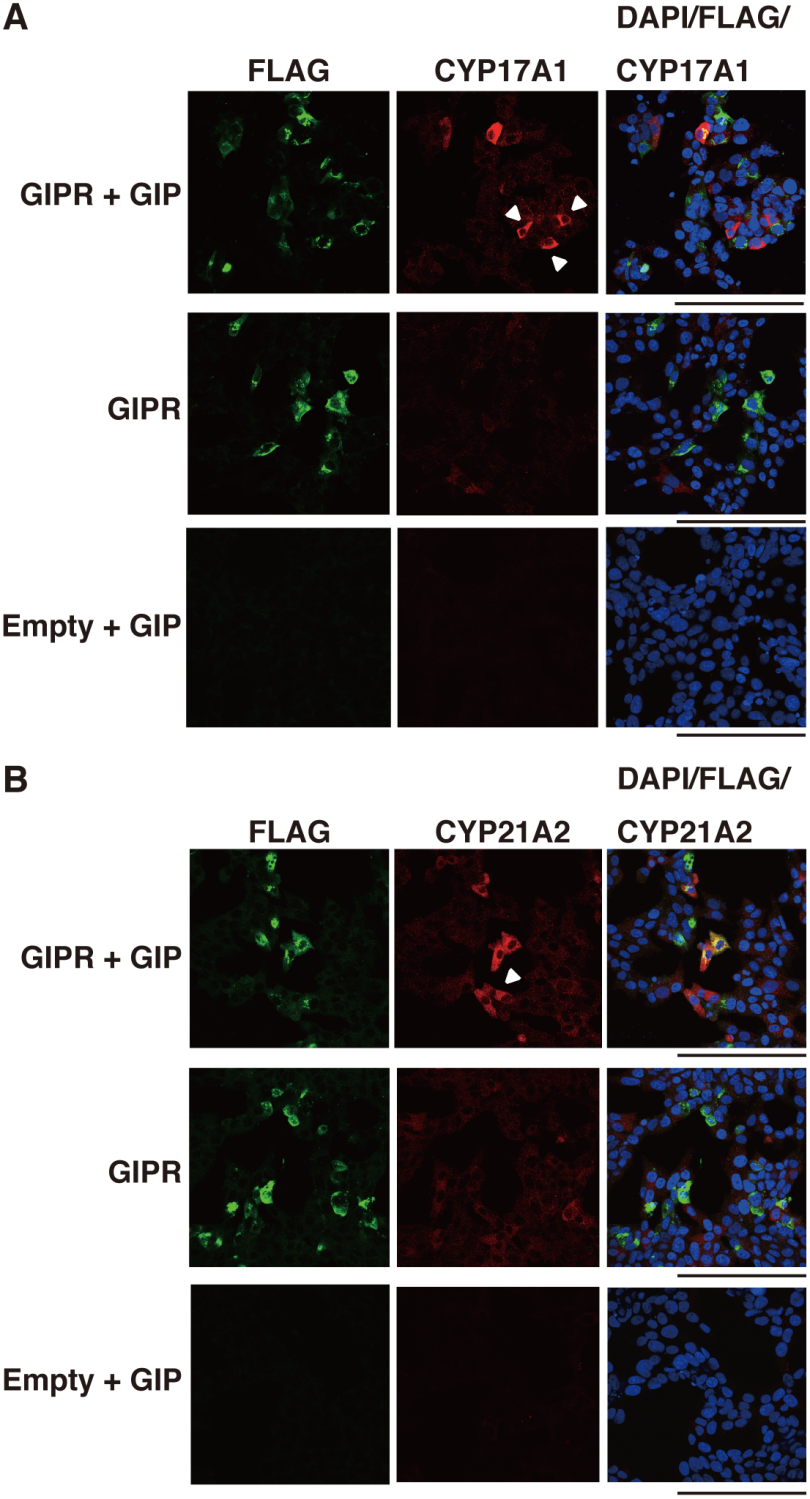
Effects of GIPR activation on the expression of CYP17A1 and CYP21A2. H295R cells were transiently transfected with the empty vector or human GIPR expression vector. After 48 h, the cells were treated with or without GIP (10^−7^ M) for 48 h under growth conditions, and fixed with 4% paraformaldehyde. (A) Immunostaining for CYP17A1. Red staining shows the anti-CYP17A1 antibody, green staining shows the anti-FLAG antibody and blue staining shows DAPI (cell nuclei). Arrowheads show FLAG-negative and CYP17A1-positive cells. (B) Immunostaining for CYP21A2. Red staining shows the anti-CYP21A2 antibody, green staining shows the anti-FLAG antibody and blue staining shows DAPI (cell nuclei). Arrowheads show FLAG-negative and CYP21A2-positive cells. Scale bars represent 100 µm.

Then, the cells were double-stained with antibodies against FLAG and CYP17A1 or CYP21A2. In the cells transfected with the empty vector treated with or without GIP, there were no FLAG-, CYP17A1-, or CYP21A2-positive cells. In the cells transfected with GIPR, but not with GIP, there were some FLAG-positive [FLAG (+)] cells, but they expressed neither CYP17A1 (Fig. 4A) nor CYP21A2 (Fig. 4B). In the GIPR-transfected and GIP-treated cells, we observed some FLAG (+) cells expressing CYP17A1 (Fig. 4A) and CYP21A2 (Fig. 4B). Interestingly, these enzymes were also expressed in some FLAG-negative [FLAG (–)] cells adjacent to the FLAG (+) cells (arrowheads, Fig. 4A and 4B), indicating that steroidogenic enzyme expression is not specific to cells expressing GIPR. Furthermore, the concentration of cortisol in the medium increased 1.5-fold in hGIPR cells treated with GIP compared to those with vehicle alone (Fig. 3B). These results indicate that cortisol production is dependent on the GIP-GIPR axis. It is speculated that a factor secreted from the GIPR-expressing and GIP-treated cells mediates steroidogenesis in the neighboring GIPR-non-expressing cells in an autocrine/paracrine manner.

### Promotion of ACTH secretion by GIPR activation leading to steroidogenesis via MC2R in H295R cells

**Figure 5:**
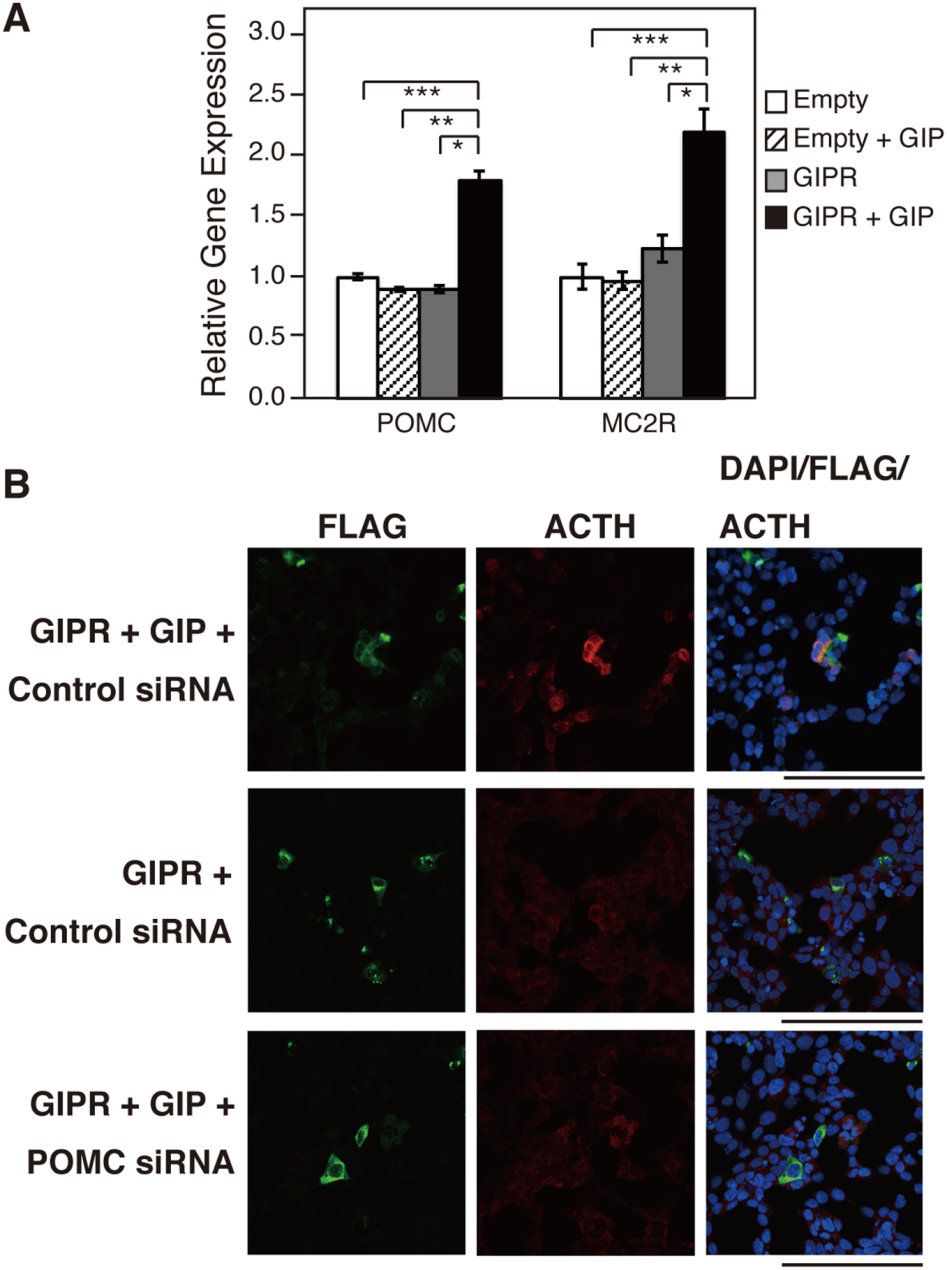
Effects of GIPR activation on the expression of ACTH. (A) Upregulation of POMC and MC2R gene expression by GIPR treated with GIP. H295R cells were treated and RNA was then extracted as in Figure 3A. The relative mRNA expression of the indicated genes was analyzed by qRT-PCR. Data are presented as mean ± SE of three independent experiments. *P<0.05 vs. GIPR + vehicle, **P<0.05 vs. empty + GIP, ***P<0.05 vs. empty + vehicle. (B) Immunostaining for ACTH (1–39). Red staining shows the anti-ACTH (1–39) antibody, green staining shows the anti-FLAG antibody and blue staining shows DAPI (cell nuclei). H295R cells were co-transfected with indicated siRNAs and the human GIPR expression vector. After 48 h, the cells were treated with or without GIP (10^−7^ M) for 48 h in growth medium, and fixed with 4% paraformaldehyde.

Having obtained evidence for the presence of a steroidogenesis-inducing factor secreted from GIP-stimulated and GIPR-expressing cells, we investigated whether ACTH, the most important steroidogenesis-inducing factor in adrenocortical cells, is produced and secreted by GIP-GIPR H295R cells. The prohormone POMC undergoes post-translational processing to ACTH before secretion into the systematic circulation. As shown in Fig. 5A, POMC mRNA level was clearly upregulated in GIPR-expressing H295R stimulated by GIP. ACTH acts by binding to a specific cell surface ACTH receptor (MC2R). MC2R mRNA level was increased 2-fold. Moreover, elevation in ACTH protein level was also observed in some FLAG (+) as well as FLAG (–) cells adjacent to the FLAG (+) cells of GIP-treated H295R-GIPR cells using immunofluorescence (Fig. 5B).

**Figure 6:**
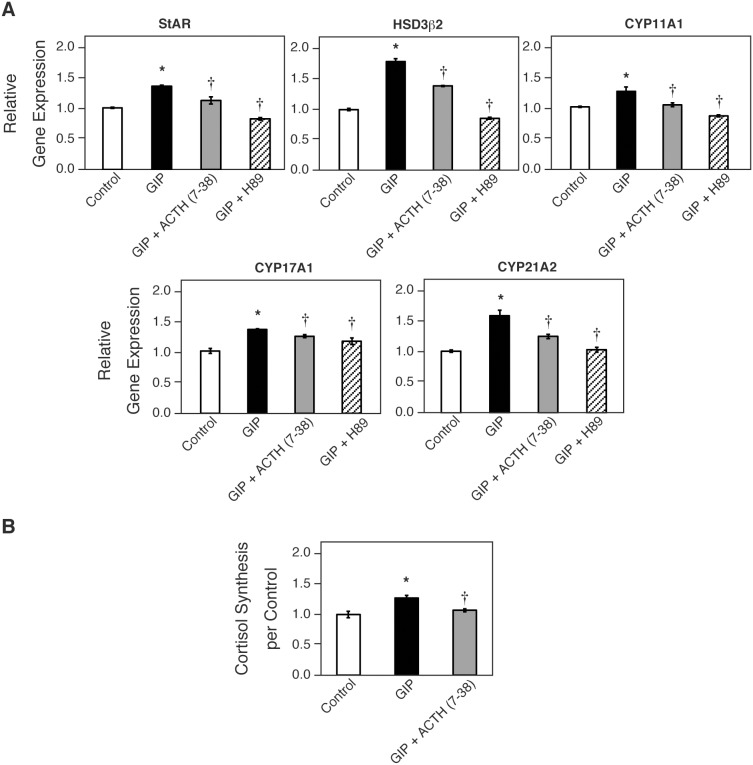
Inhibitory effect of ACTH (7–38) on GIPR-stimulated steroidogenesis. At 1 h before GIP stimulation, GIPR-transfected H295R cells were treated with or without ACTH (7–38) (10^−7^ M), or H89 (10 µM), and then incubated with GIP for 24 h in starvation medium (A), or for 48 h in growth medium (B). (A) Relative mRNA expression of the indicated genes was analyzed by qRT-PCR. Data are presented as mean ± SE of three independent experiments. (B) Cortisol concentration in the medium was measured using ELISA. Data are presented as mean ± SE of three independent experiments. *P<0.05 vs. vehicle, †P<0.05 vs. GIP.

**Figure 7:**
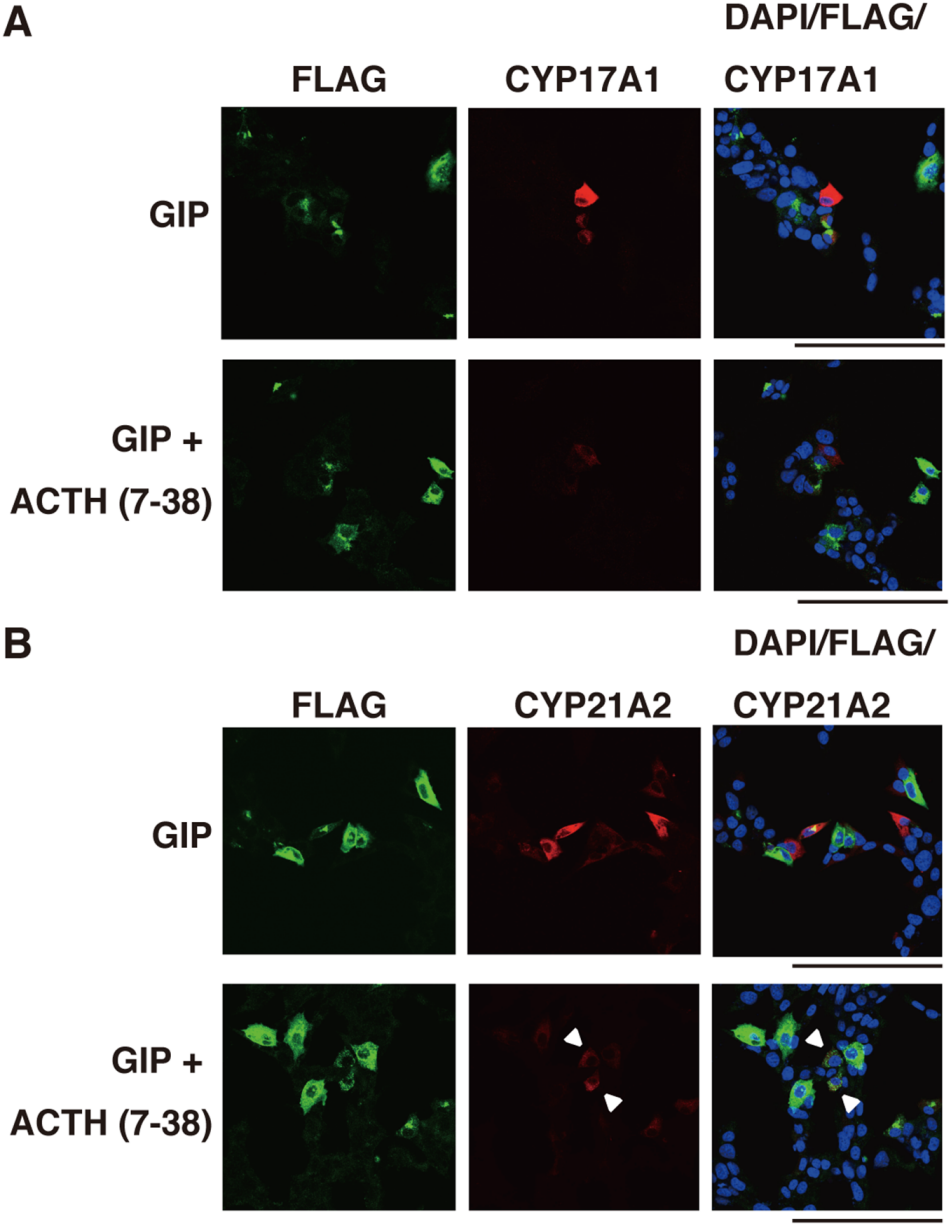
Inhibitory effect of ACTH (7–38) on the expression of CYP17A1 and CYP21A2 promoted by GIP. At 1 h before GIP stimulation, GIPR-transfected H295R cells were treated with or without ACTH (7–38) (10^−7^ M), and then incubated with GIP for 48 h. (A) Immunostaining for CYP17A1. Red staining shows the anti-CYP17A1 antibody, green staining shows the anti-FLAG antibody and blue staining shows DAPI (cell nuclei). (B) Immunostaining for CYP21A2. Red staining shows the anti-CYP21A2 antibody, green staining shows the anti-FLAG antibody and blue staining shows DAPI (cell nuclei). Scale bars represent 100 µm.

**Figure 8:**
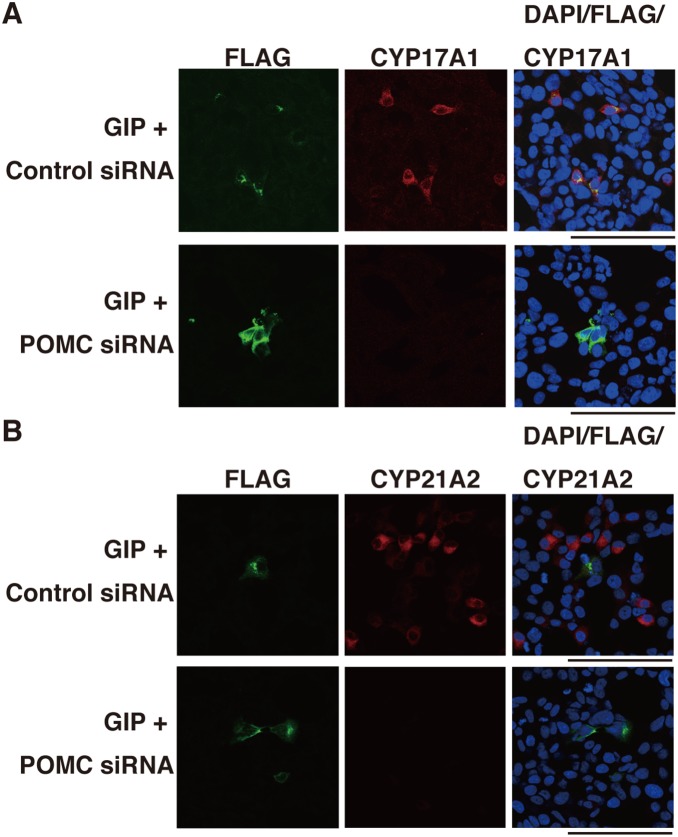
Inhibitory effect of POMC siRNA on the expression of CYP17A1 and CYP21A2 promoted by GIP. H295R cells were treated and fixed as in Figure 5B. (A) Immunostaining for CYP17A1. Red staining shows the anti-CYP17A1 antibody, green staining shows the anti-FLAG antibody and blue staining shows DAPI (cell nuclei). (B) Immunostaining for CYP21A2. Red staining shows the anti-CYP21A2 antibody, green staining shows the anti-FLAG antibody and blue staining shows DAPI (cell nuclei). Scale bars represent 100 µm.

To investigate the role of secreted ACTH in steroidogenesis, human corticotropin inhibiting peptide [corticotropin (7–38); Wako] was used as a substance with respect to its antagonistic action toward the MC2R. GIP-stimulated StAR, HSD3β2, CYP11A1, CYP17A1, and CYP21A2 mRNA transcripts were partially inhibited by ACTH (7–38), whereas H89, a PKA inhibitor, completely inhibited their expression (Fig. 6A). Immunofluorescence analysis showed that the expression of CYP21A2 and CYP17A1 was remarkably reduced in cells treated with both GIP and ACTH (7–38) compared with cells treated with GIP alone (Fig. 7A and 7B). Noticeably, the expression of those molecules remained weakly only in FLAG (+) cells (arrowheads, Fig. 7B), whereas it was completely inhibited by H89 treatment (data not shown). ACTH (7–38) also repressed cortisol synthesis induced by GIPR activation (Fig. 6B). We further investigated the role of POMC in steroidogenesis in GIP-GIPR H295R cells. As shown in Figure 8, POMC inactivation by siRNA in H295R cells suppressed CYP17A1 and CYP21A2 expression at the single cell level (Fig. 5B, 8 and Fig. S2).

**Figure 9:**
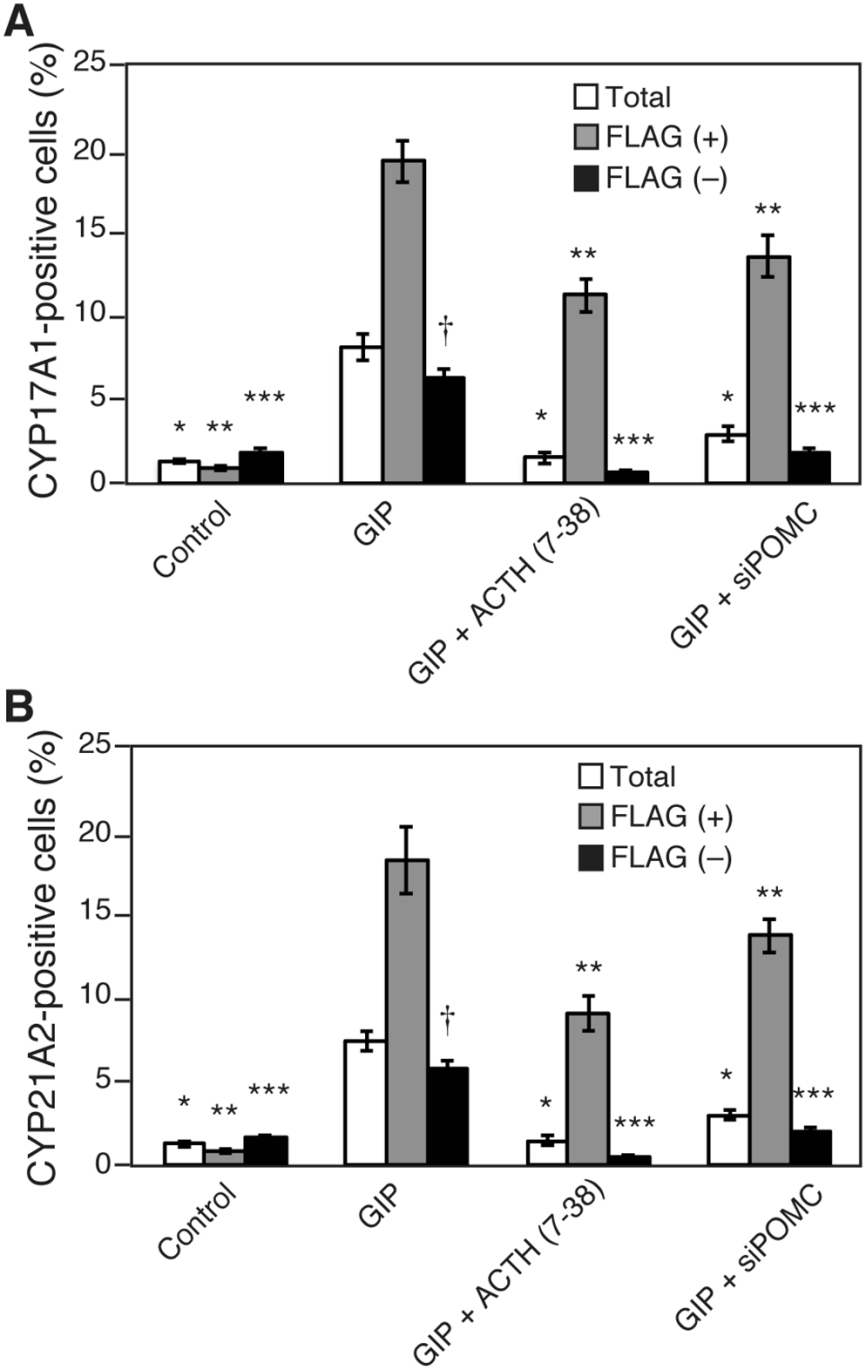
Quantitative analysis of steroidogenic enzyme-positive cells in immunofluorescence experiments. H295R cells were treated as Figure 4, 7, or 8, and fixed with 4% paraformaldehyde, followed by immunofluorescence. The percentages of steroidogenic enzyme-positive cells in total cells, FLAG-positive or -negative cells were measured. At least 200 cells from random fields in a blinded manner were scored for each condition. Data are presented as mean ± SE of three independent experiments. *P<0.05 vs. total cells treated with GIP, **P<0.05 vs. FLAG (+) cells treated with GIP, ***P<0.05 vs. FLAG (–) cells treated with GIP, †P<0.05 vs. FLAG (+) cells treated with GIP. (A) CYP17A1, (B) CYP21A2.

Finally, we performed quantitative analysis of steroidogenic enzyme-positive cells in FLAG-tagged GIPR-transfected cells. The rate of steroidogenic enzyme-positive cells was increased both in FLAG (+) and FLAG (–) cells (Fig. 9). Of note, number of fold increase was significantly larger in FLAG (+) than in FLAG (–) cells. Treatment with ACTH (7–38) as well as with siPOMC reduced the number of steroidogenic enzyme-positive cells to the control level in FLAG (–) cells, but such reduction was partial in FLAG (+) cells, suggesting the presence of ACTH-independent steroidogenic mechanism in these populations. On the other hand, in cells transfected with empty vector containing no GIPR gene did not yield any steroidogenic enzyme-positive cells after GIP treatment (Fig. 4). These data indicate that steroidogenic enzyme expression could occur in a GIP-GIPR axis-dependent manner. Further, in FLAG (–) cells located adjacent to FLAG (+), steroidogenesis seems to occur by cell-intrinsic ACTH-MC2R system geared by a GIP-GIPR axis that emerged in neighboring GIPR (+) cells. In contrast, in FLAG (+) cells, steroidogenesis is triggered both by ACTH-dependent autocrine and ACTH-independent mechanisms.

## Discussion

In this study, we elucidated the mechanisms regulating steroidogenesis promoted by GIP-GIPR in human adrenal H295R cells, and conclude that GIPR activation provoked steroidogenesis via the secretion of ACTH in autocrine and paracrine manners.

Cyclic AMP leads to the activation of kinases that phosphorylate steroidogenic transcription factors, the induction of steroidogenic enzyme expression, and subsequently steroidogenesis. ACTH, the key hormone regulating glucocorticoid and androgen biosynthesis in the adrenal cortex, exerts its effects via the GPCR, MC2R which predominantly activates the second messenger cAMP [3]–[11]. The ectopic expression of other GPCRs in the adrenal glands, such as GIPR, is also known to induce steroidogenesis through cAMP activation [12]–[19]. We showed that an analog of cAMP, 8-Br-cAMP, and forskolin, which elevates cAMP via adenylate cyclase, induced steroidogenesis in H295R cells. Udhane *et al*. recently reported that 8-Br-cAMP promoted HSD3β2 expression and the synthesis of steroid hormones in H295R cells [37]. We confirmed that the mRNA expression of steroidogenic factors and enzymes including HSD3β2 was upregulated; CYP17A1 and CYP21A2 expression increased approximately 1.5-2-fold. The cortisol production increased to approximately 4-fold. Further, immunofluorescence experiments revealed that CYP17A1 and CYP21A2 accumulated in approximately 30% of the cells treated with 8-Br-cAMP and forskolin (Figure 2). Bates *et al.* reported that GIP activates steroidogenic gene expression in Y1 mouse adrenocortical cells stably expressing GIPR [27]. On the basis of these data, we investigated steroidogenesis in GIP-stimulated and GIPR-expressing H295R cells. The level of steroidogenesis-related genes was increased slightly, but significantly, and the production of cortisol increased 1.5-fold. Given our low transfection efficiency (5%), these numbers seem to be not so small. Then, in order to prove the likely connection between GIP-GIPR and expression of steroidogenic enzymes required for the production of cortisol at a single cell level more precisely, we carried out subsequent immunofluorescent microscopic experiments. Fig. 4 showed that not only FLAG (+) cells but also FLAG (–) cells expressed CYP17A1 and CYP21A2. The FLAG (–) cells expressing these enzymes were adjacent to FLAG (+) cells. Conversely, in GIPR-expressing H295R cells without GIP treatment, there were no steroidogenic enzyme-expressing cells. These data indicate that GIP-GIPR cells substantially contribute to steroidogenesis in adjacent cells. Presumably, GIP-GIPR cells may secrete a secondary factor to stimulate steroidogenesis in neighboring cells through a paracrine mechanism.

What is the secondary factor? The first candidate factor to mediate steroidogenesis is ACTH. ACTH is a 39 amino acid polypeptide that is synthesized predominantly in and secreted from the anterior lobe of the pituitary gland. The synthesis and secretion of ACTH are tightly controlled by the hypothalamic-pituitary-adrenal axis. Under stress conditions, the paraventricular nucleus of the hypothalamus secretes corticotropin-releasing hormone (CRH). CRH regulates the anterior lobe of the pituitary gland and stimulates the secretion of ACTH. Additionally, various factors such as IL-6, LIF, arginine-vasopressin, and oncostatin M induce ACTH synthesis and secretion [38]–[42]. In the adrenal glands, ACTH acts by binding to a specific cell surface ACTH receptor, MC2R. MC2R is a seven-membrane-spanning GPCR that is primarily expressed in adrenocortical cells. Upon ligand binding, the receptor undergoes conformational changes that stimulate adenylyl cyclase, leading to an increase in intracellular cAMP, the activation of PKA, and subsequent steroidogenesis. ACTH is synthesized and secreted by not only pituitary corticotroph cells but also by cells such as lymphocytes and chromaffin cells.

Of particular interest, Louiset *et al*. recently reported that corticotropin (ACTH) is produced by subpopulation of steroidogenic cells in the hyperplastic adrenal glands of patients with macronodular hyperplasia, and the secreted ACTH controls subsequent steroidogenesis [20]. The hyperplastic adrenal samples they examined consisted of heterogeneous cell lineages. Even if cells in the adrenocortical samples express steroidogenic factors such as SF1, these cells could have originated from different cell types. We showed the upregulation of the mRNA level of POMC, a precursor of ACTH, and MC2R in the GIP-treated and GIPR-expressing H295R adrenocortical cancer cell line. Immunofluorescence revealed that ACTH was expressed in GIP-treated H295R-GIPR cells, some FLAG (+) and FLAG (–) cells adjacent to the FLAG (+) cells. It is likely that GIP-activated GIPR mediates synthesis and secretion of ACTH, which induces further ACTH synthesis as well as steroidogenesis in adrenocortical cells.

We investigated whether secreted ACTH is involved in steroidogenesis by activated GIPR. The GIP-induced secretion of cortisol was partially reduced by the MC2R antagonist ACTH (7–38), which is a fragment of corticotropin corresponding to amino acids 7 to 38 of the peptide. Further, siRNA against POMC inhibited steroidogenesis in a manner similar to ACTH (7–38). These results indicate that the activated GIPR-mediated steroidogenesis occurs, at least in part, through an interaction between ACTH and MC2R. It is noted that H89, an inhibitor of PKA, completely inhibited the mRNA expression of steroidogenic factors, whereas its inhibition by ACTH (7–38) was partial. Immunofluorescence analysis revealed that after treatment with ACTH (7–38), CYP17A1 and CYP21A2 disappeared largely in FLAG (–) cells, while they remained partially in FLAG (+), GIPR-expressing cells (Fig. 9). These indicate that while steroidogenesis is promoted mainly by ACTH-MC2R axis in FLAG (–) cells, GIP-GIPR partially activates steroid production independently of ACTH-MC2R axis in FLAG (+) cells. In GIP-treated GIPR-introduced cells, we measured ACTH concentration in the medium by electrochemiluminescence immunoassay (ECLIA), which revealed no significant increase in ACTH secretion in the culture medium. Nonetheless, immunofluorescence clearly showed expression of cellular ACTH protein in some GIP-treated GIPR-introduced cells, which was inhibited by introduction of POMC siRNA (Fig. 5B). We speculate that rather minute amounts of ACTH are released locally from GIPR (+) cells after GIP treatment and act in a paracrine/autocrine fashion.

These observations are consistent with the notion that a process mediated via GIPR, and activated by a factor other than ACTH, is surely present. Thus another receptor, for example, the receptor of corticotropin releasing hormone (CRH), the luteinizing hormone receptor (LHR) and other G protein coupled receptors should be considered. In this paper, we did not check expression of other receptors in H295R cells, and are not able to deny the possibility that another receptor located on H295R cells that could be stimulated by GIP, is able to regulate CYP17A1 and CYP21A2 expression. The involvement of other receptors would be investigated in future study.

In this study, we used serum-free medium in qRT-PCR experiments, and serum-containing medium in immunofluorescence and cortisol assay. First, we performed all experiments under starved condition, because serum includes many factors, which may affect cortisol synthesis and secretion. However, in cortisol assay, the concentration of cortisol in the medium of control, and GIP-GIPR-, forskolin- or 8-bromo cAMP-stimulated cells was below the measurable limit in starved condition. In growth condition, secretion of cortisol was clearly stimulated by GIP-GIPR, forskolin or 8-bromo cAMP, but not in the control medium. In immunofluorescence, CYP17A1 and CYP21A2 were expressed in stimulated H295R cells in both medium conditions, but the level of expression (but not rate of these enzyme-positive cells) was slightly higher in growth condition rather in starved condition, indicating that serum may affect CYP17A1 and CYP21A2 protein stability. Overall, these suggest that downstream pathway of CYP17A1 and CYP21A2 protein synthesis in cortisol production may need synergistically some factor(s) in the serum to stimulate cortisol secretion.

Our *in vitro* study provides direct evidence that steroidogenesis mediated by activated GIPR is controlled by GIP-GIPR via MC2R-ACTH neo-axis- dependent or independent mechanism using immunofluorescent microscopic experiments at the single cell level. It is reported that ectopic expression of GIPR in adrenal gland induces steroidogenesis, resulting in FD-CS [14], [15], [17]–[19]. Indeed, by immunofluorescence, we detected GIPR in specimens of adrenocortical tumor obtained from a patient with FD-CS one of us (Y.T.) and his colleagues had reported previously [43] (Fig. S3A). We also found that cultured cells derived from the specimen secreted cortisol after treatment with GIP but not a vehicle (Fig. S3B). These suggest that GIPR is not only ectopically expressed in the adrenocortical tumor, but also is activated by GIP after each meal, resulting in periodic cortisol secretion. Thus our *in vitro* experiments might well reflect the pathological condition of the patient’s adrenal gland.

Here, we have focused on the relationship between GIPR and steroidogenesis, but we believe that the principles of our assay are generalizable to other factors including GPCRs. In recent years, genomic research on adrenocortical tumors has yielded a number of new findings; comprehensive data on gene and miRNA expression, the genome, and methylation alterations in adrenocortical carcinomas are now available [44]. In the next step, to evaluate the role of the newly identified factors in the pathophysiology of adrenocortical tumors, our single cell analysis on double immunostaining of the factor with not only steroidogenic related factors, but also a marker of proliferation (e.g. Ki67) using H295R cells transiently expressing the factor might be helpful. Thus, our approach has the potential to provide clues as to the underlying molecular mechanisms of adrenocortical tumors.

In summary, we showed that GIP activates steroidogenesis in GIPR-expressing H295R adrenocortical cells through the expression of genes for steroidogenic factors and enzymes and the accumulation of their respective proteins. Steroidogenesis proceeds via two pathways, the ACTH-MC2R and GIP-GIPR systems. These results were confirmed by immunofluorescent microscopic experiments at the single cell level. This study provides information that may be helpful in the elucidation of the pathology, subsequent development of therapy, and maintenance of adrenocortical tumors.

## Supporting Information

Figure S1 **Expression of FLAG/GIPR in H295R cells transfected with human FLAG-tagged GIPR gene.** H295R cells were transiently transfected with the empty vector or human GIPR expression vector. Immunostaining for FLAG and GIPR. Green staining shows the anti-FLAG antibody, red staining shows the anti-GIPR antibody, and blue staining shows DAPI (cell nuclei). Scale bars represent 100 µm.
(TIF)

Figure S2 **Inhibitory effect of POMC siRNA on the expression of POMC.** Relative mRNA expression of POMC gene was analyzed by qRT-PCR. H295R cells were transfected with the indicated siRNA. At 24 h after transfection, the culture medium was changed to the starvation medium. After 24 h, the cells were treated with GIP (10^−7^ M) for 24 h, and following this, RNA was extracted. Data are presented as mean ± SE of three independent experiments. *P<0.05 vs. control siRNA.
(TIF)

Figure S3 **Analysis of adrenal gland tumor samples from a patient with FD-CS.** (A) GIPR expression in samples of adrenal gland tumor from a patient with FD-CS. Normal portion in adrenal gland from a patient with aldosterone-producing adrenal tumor was used as a control. Immunostaining for GIPR and CYP21A2. Green staining shows the anti-GIPR antibody, red staining shows the anti-CYP21A2 antibody, and blue staining shows DAPI (cell nuclei). Scale bars represent 100 µm. (B) GIP stimulated cortisol production in cultured cells derived from an adrenal tumor specimen of a patient with FD-CS. The cells were treated with GIP (0, 0.2, 2.0 or 20 nM) for 24 h. Cortisol concentration of the culture medium was measured using ELISA. *P<0.05 vs. GIP 0.0 (nM).
(TIF)

Table S1 **Primer sequences for quantitative RT-PCR.**
(DOCX)

File S1 **Methods for experiments of patients’ samples (Figure S3).**
(DOCX)
