# Supplementary figures and images for: Marked Cortisol Production by Intracrine ACTH in GIP-Treated Cultured Adrenal Cells in Which the GIP Receptor Was Exogenously Introduced
Source: PLoS One. 2014 Oct 21;9(10):e110543. doi: 10.1371/journal.pone.0110543 (PMC4204891; doi:10.1371/journal.pone.0110543)

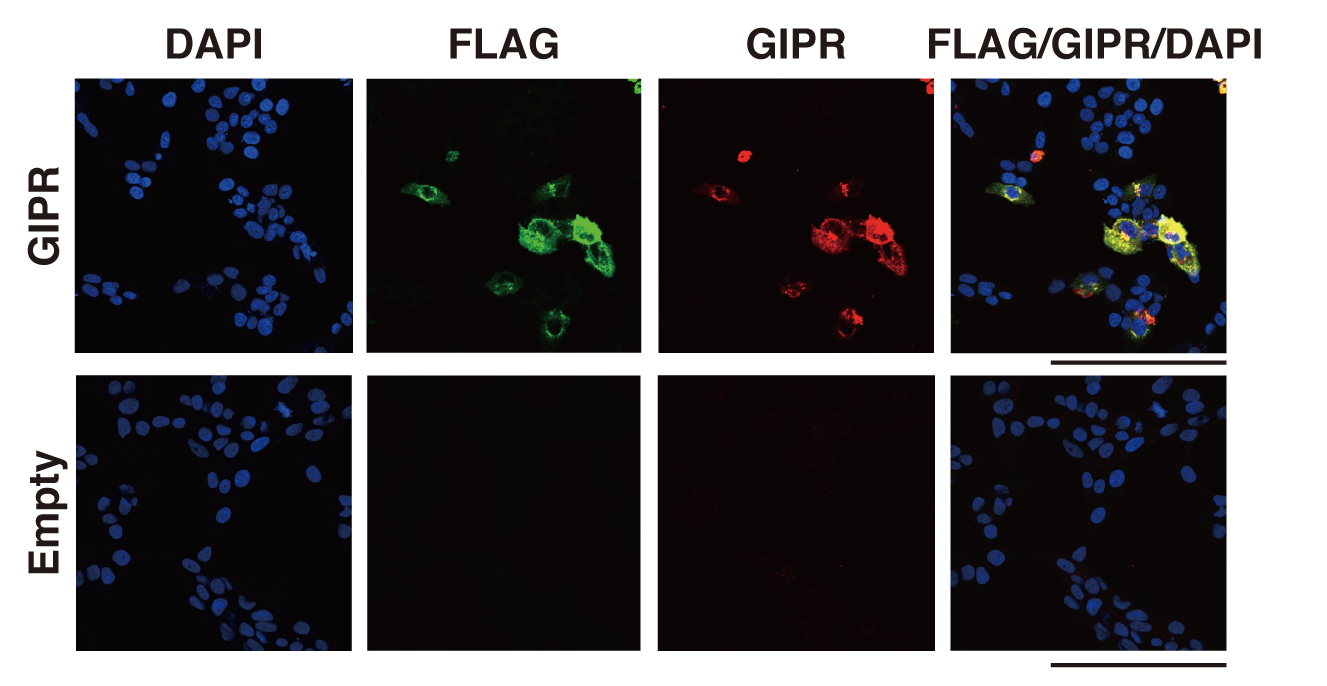

Supplement: Figure S1 — Expression of FLAG/GIPR in H295R cells transfected with human FLAG-tagged GIPR gene. H295R cells were transiently transfected with the empty vector or human GIPR expression vector. Immunostaining for FLAG and GIPR. Green staining shows the anti-FLAG antibody, red staining shows the anti-GIPR antibody, and blue staining shows DAPI (cell nuclei). Scale bars represent 100 µm. (TIF) [file pone.0110543.s001.tif]

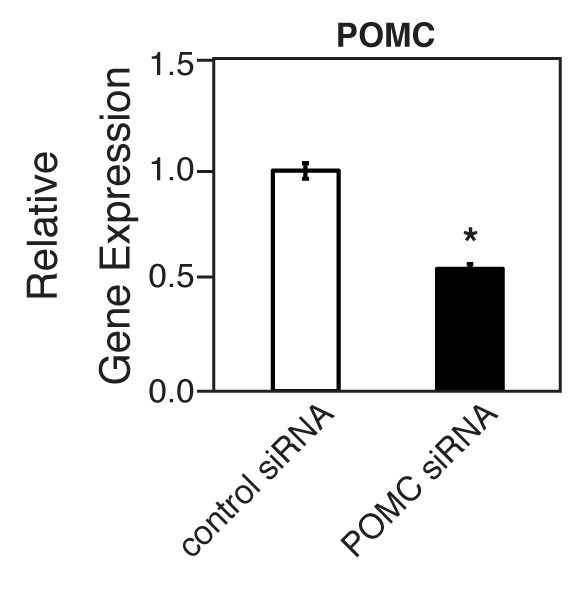

Supplement: Figure S2 — Inhibitory effect of POMC siRNA on the expression of POMC. Relative mRNA expression of POMC gene was analyzed by qRT-PCR. H295R cells were transfected with the indicated siRNA. At 24 h after transfection, the culture medium was changed to the starvation medium. After 24 h, the cells were treated with GIP (10−7 M) for 24 h, and following this, RNA was extracted. Data are presented as mean ± SE of three independent experiments. *P<0.05 vs. control siRNA. (TIF) [file pone.0110543.s002.tif]

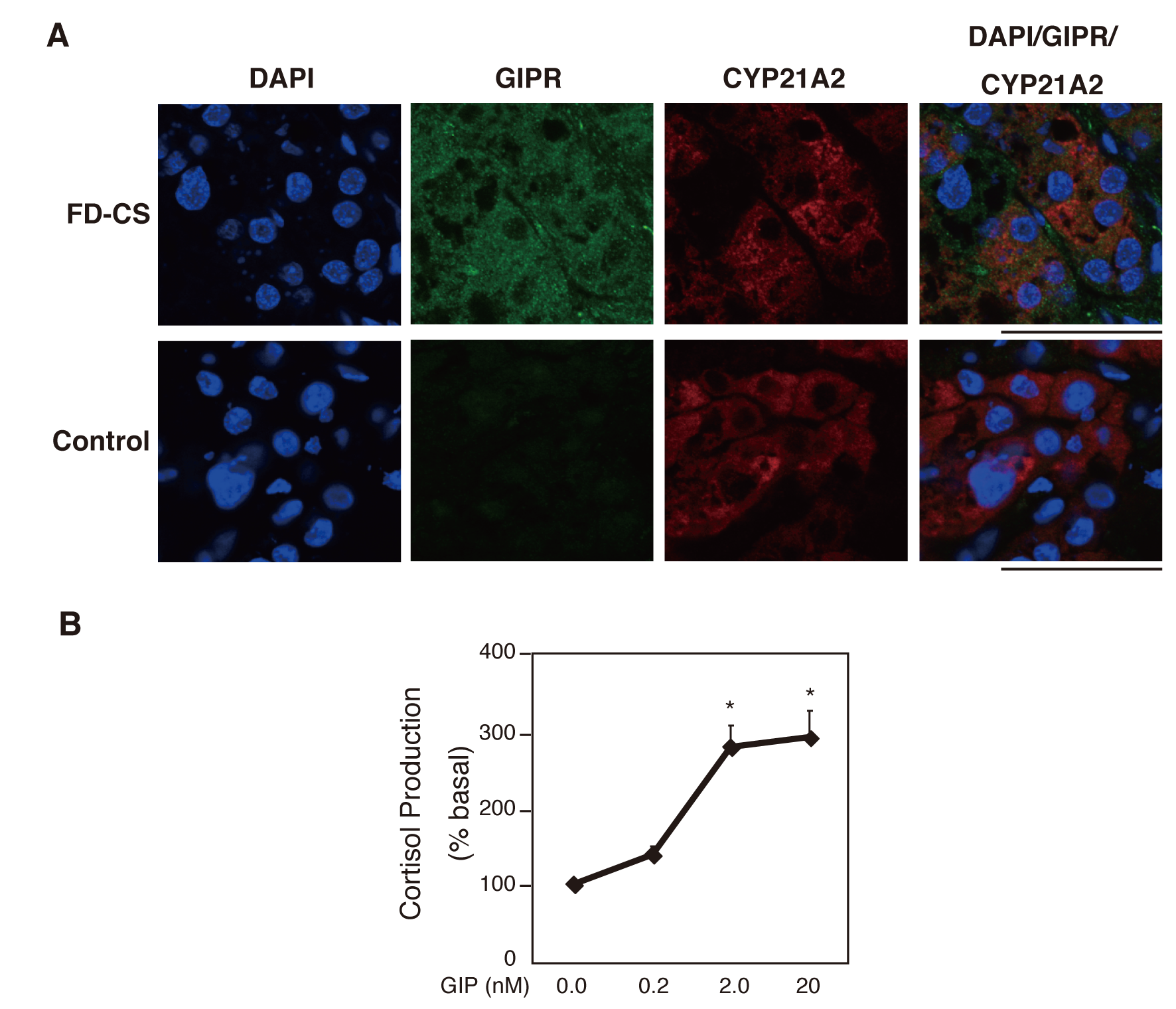

Supplement: Figure S3 — Analysis of adrenal gland tumor samples from a patient with FD-CS. (A) GIPR expression in samples of adrenal gland tumor from a patient with FD-CS. Normal portion in adrenal gland from a patient with aldosterone-producing adrenal tumor was used as a control. Immunostaining for GIPR and CYP21A2. Green staining shows the anti-GIPR antibody, red staining shows the anti-CYP21A2 antibody, and blue staining shows DAPI (cell nuclei). Scale bars represent 100 µm. (B) GIP stimulated cortisol production in cultured cells derived from an adrenal tumor specimen of a patient with FD-CS. The cells were treated with GIP (0, 0.2, 2.0 or 20 nM) for 24 h. Cortisol concentration of the culture medium was measured using ELISA. *P<0.05 vs. GIP 0.0 (nM). (TIF) [file pone.0110543.s003.tif]
