# Supplementary material for: Marked Cortisol Production by Intracrine ACTH in GIP-Treated Cultured Adrenal Cells in Which the GIP Receptor Was Exogenously Introduced
Source: PLoS One. 2014 Oct 21;9(10):e110543. doi: 10.1371/journal.pone.0110543 (PMC4204891; doi:10.1371/journal.pone.0110543)
Supplement: Table S1 — Primer sequences for quantitative RT-PCR. (DOCX) [file pone.0110543.s004.docx]

**Table S1**

**Primer sequences for quantitative RT-PCR**

| Gene |  | Primer sequence |
| --- | --- | --- |
| StAR | Forward | 5' -GCATCGGTGAGTTTGCTGTG- 3' |
|  | Reverse | 5' -GATTCAAGAAACGCTCAGC- 3' |
| HSD3β2 | Forward | 5' -GCCTGTTGGTGGAAGAGAAG- 3' |
|  | Reverse | 5' -ATGATACAGGCGGTGTGGAT- 3' |
| CYP11A1 | Forward | 5' -GGAAATTACTCGGGGGACAT- 3' |
|  | Reverse | 5' -CACATGGTCCTTCCAGGTCT- 3' |
| CYP17A1 | Forward | 5' -CTCTCTACTCGGTTCTCGGC- 3' |
|  | Reverse | 5' -GGACACCTTGCCCACATCT- 3' |
| CYP21A2 | Forward | 5' -AACTACCCGGACCTGTCCTT- 3' |
|  | Reverse | 5' -TCTCATGCGCTCACAGAACT- 3' |
| POMC | Forward | 5' -CAGCCAGTGTCAGGACCTC- 3' |
|  | Reverse | 5' -GGTCAGAGGCTGCTCGTC- 3' |
| MC2R | Forward | 5' -GGACCGCTACATCACCATCT- 3' |
|  | Reverse | 5' -AGGCACAGGATGAAGACCAG- 3' |
| GAPDH | Forward | 5' -GAGCCAAAAGGGTCATCATC- 3' |
|  | Reverse | 5' -CCATCCACAGTCTTCTGGGT- 3' |
