# Supplementary material for: Marked Cortisol Production by Intracrine ACTH in GIP-Treated Cultured Adrenal Cells in Which the GIP Receptor Was Exogenously Introduced
Source: PLoS One. 2014 Oct 21;9(10):e110543. doi: 10.1371/journal.pone.0110543 (PMC4204891; doi:10.1371/journal.pone.0110543)
Supplement: File S1 — Methods for experiments of patients’ samples (Figure S3). (DOCX) [file pone.0110543.s005.docx]

**Methods for experiments using patients’ samples.**

We obtained specimens of adrenal gland tumor from a patient with FD-CS, and normal lesion in adrenal gland from a patient with aldosterone-producing adrenal gland tumor as a control. Specimens were collected at surgery from patients.

***Tissue specimens***

Tissue samples of the adrenals from patients were embedded in Tissue-Tek O.C.T. Compound (Sakura Finetek Japan, Tokyo, Japan) in liquid nitrogen. Seven-micrometer-tissue sections were cut using cryomicrotome (Leica CM3050S, Microsystems, Nussloch, Germany) at −21°C.

***Immunofluorescence***

For fixation of tissues, 4% paraformaldehyde in phosphate-buffered saline (PBS) was added to the samples and incubated for 20 min at room temperature. Permeabilization was carried out with 0.2% Triton X-100 in PBS for 20 min at room temperature. Nonspecific binding was blocked by incubation in 4% bovine serum albumin, 2% donkey serum, and 0.1% Triton X-100 in PBS for 1h at room temperature. Anti-rabbit GIPR monoclonal antibody and anti-goat CYP21A2 polyclonal antibody were diluted in the above blocking solution at the concentration of 1: 50, and incubated overnight at 4°C. Secondary antibodies, Alexa Fluor 546 donkey anti-rabbit IgG (H+L) (for GIPR detection) and Alexa Fluor 647 donkey anti-goat IgG (H+L) (for CYP21A2 detection) were also diluted in blocking solution at the concentration of 1: 500, and incubated for 30 min at 37°C. Nuclei were stained with 2 μg/ml DAPI (4’6-Diamidine-2’-phenylindole dihydrochloride) in PBS for 15 min. Images were acquired using the laser-scanning confocal image system (A1R-A1 Confocal Microscope System) (Nikon, Japan).

***Adrenal Cell culture***

Adrenal gland fragments were immersed in culture medium (50% DMEM to 50% Ham’s F-12) supplemented with 0.2% antibiotic-antimycotic solution and rapidly transported to the laboratory. The fragments were minced with scissors, and adrenal cells were enzymatically dispersed, as described [1, 2]. Briefly, tissue samples were stirred for 45 min at 37°C in culture medium containing protease (2 mg/ml), collagenase (2 mg/ml), and deoxyribonuclease I (70 μg/ml) in a 5% CO_2_-95% air atmosphere. The tissues were disaggregated by gentle aspirations with a sterile 10 ml pipette. Dispersed cells were filtered on a nylon sieve (100-μm mesh opening). The cell suspension was centrifuged (100 X g, 37°C, 20 min). Residual tissue fragments were subjected to a second period of digestion/dispersion procedure as described above. Isolated cells were then transferred into culture medium supplemented with 5 μg/ml insulin, 10 μg/ml apotransferrin, 20 μg/ml ascorbic acid, and 5% fetal calf serum. Cells were cultured in 6-well dishes (at a density of 2 X 10^5^ cells per well) and incubated at 37°C in a 5% CO_2_-95% air atmosphere with 100% relative humidity. The culture medium was changed 24 h after plating. Incubation experiments of cells were conducted after 2 d in culture with fresh DMEM (control) or DMEM containing different concentration of GIP. Cells were incubated for 24 h. After incubation period, aliquots of the culture medium were taken and immediately frozen at -80°C until cortisol assay.

***References***

1. Louiset E, Duparc C, Young J, Renouf S, Tetsi Nomigni M, et al. (2013) Intraadrenal corticotropin in bilateral macronodular adrenal hyperplasia. N Engl J Med 369: 2115-2125.
2. Bertherat J, Contesse V, Louiset E, Barrande G, Duparc C, et al. (2005) In vivo and in vitro screening for illegitimate receptors in adrenocorticotropin-independent macronodular adrenal hyperplasia causing Cushing's syndrome: identification of two cases of gonadotropin/gastric inhibitory polypeptide-dependent hypercortisolism. J Clin Endocrinol Metab 90: 1302-1310.
